# Sexual attitudes, pattern of communication, and sexual behavior among unmarried out-of-school youth in China

**DOI:** 10.1186/1471-2458-7-189

**Published:** 2007-07-31

**Authors:** Bo Wang, Xiaoming Li, Bonita Stanton, Vafa Kamali, Sylvie Naar-King, Iqbal Shah, Ronald Thomas

**Affiliations:** 1 Department of Community Health Sciences, the University of Southern Mississippi, Hattiesburg, MS, USA; 2 The Carman and Ann Adams Department of Pediatrics Prevention Research Center, Wayne State University, Detroit, MI, USA; 3 Department of Reproductive Health and Research, World Health Organization, Geneva, Switzerland

## Abstract

**Background:**

In recent years, more adolescents are engaging in premarital sex in China. However, only a limited number of studies have explored out-of-school youth's sexual attitudes and behaviors, critical for prevention intervention development.

**Methods:**

Using data from the baseline survey of a comprehensive sex education program that was conducted in a suburb of Shanghai in 2000–2002, this study describes sexual attitudes, patterns of communication on sexual matters, and premarital sexual behavior among 1,304 out-of-school youth. Multivariate logistic regression analysis was conducted to examine the factors associated with youth's premarital sexual intercourse.

**Results:**

The majority (60%) of out-of-school youth held favorable attitudes towards premarital sex. Males were more likely to have favorable attitudes compared with females. Male youth generally did not communicate with either parent about sex, while one-third of female youth talked to their mothers about sexual matters. Both males and females chose their friends as the person with whom they were most likely to talk about sexual matters. About 18% of the youth reported having engaged in sexual intercourse. One-fifth of sexually active youth had always used a contraceptive method, and one-quarter had been pregnant (or had impregnated a partner). There were no gender differences in rate of premarital sex or frequency of contraceptive use. Multivariate analysis revealed that age, education, family structure, parent's discipline, attitudes towards premarital sex, pattern of communication and dating were significantly associated with youth premarital sex.

**Conclusion:**

A substantial proportion of out-of-school youth engage in risky sexual behaviors. Prevention programs that empower communication and sexual negotiation skills, and promote condom use should be implemented for this vulnerable group.

## Background

Over the last two decades, China has experienced profound social changes associated with the economic reforms. Attitudes towards sex have been changing rapidly, and premarital sex has been accepted by many young people [1,2]. The prevalence of sexually transmitted infections (STIs) has increased dramatically during this period [3], and heterosexual transmission is rapidly becoming the primary route of HIV transmission in China. Sexually transmitted HIV infection in China increased from 5.5% in 1997 to 19.8% in 2003 [4]. It is estimated that half of the new HIV infections in China in 2005 occurred through unprotected sex [5].

Young people are particularly vulnerable to HIV infection. The United Nations estimated that about half of new HIV infections worldwide occur among young people ages 15–24 [5]. In China, more than 60% of all HIV infections are among people aged 15–29 years [6]. Effective HIV prevention among youth, therefore, is critical to containment of the HIV/AIDS epidemic, and understanding the sexual attitudes and behavior of youth is important to designing prevention interventions.

Previous studies have demonstrated that out-of-school status is strongly associated with increased sexual activity and sexual risk behaviors [7,8]. A study in Ethiopia reported that a considerably higher proportion of out-of-school youth than in-school youth engage in unprotected intercourse [8]. In New South Wales, out-of-school youth had consistently higher rates of sexual behavior compared to their age matched cohorts in school [9]. In the United States, out-of-school youth are more likely to initiate sex earlier, to fail to use contraception, and to become pregnant [10-12]. Out-of-school youth have higher frequencies than in-school youth of behaviors that increase risk for STIs and HIV infection [13]. A recent comprehensive review has shown that sexual risk and prevalence of STIs peaks at this age among young adults in the United States [14].

Family context has been shown to have consistent and strong effects on the timing of sexual debut. Adolescents living in single-parent families or with stepparents initiate sexual activity earlier than those in two-parent families [15,16]. Adolescent feelings of closeness and connectedness to parents, parental disapproval of sex, and positive peer influences have been shown to delay sexual activity [17,18]. Dating, and especially early steady dating, provides a context for many adolescent sexual experiences [19]. Previous studies have shown that adolescents' attitudes about sex affect adolescent sexual behavior, more permissive attitudes lead to earlier first sex [19,20]. However, few studies have explored factors associated with premarital sex in Chinese context.

In China, little is known about the sexual attitudes and behavior among out-of-school youth. Most studies investigating young people's sexual behavior have been conducted among high school and college students. These studies indicated that most young people are accepting of premarital sex, and a growing number of youth are engaging in premarital sexual activity [21,22]. For example, a survey performed in Beijing in 1989 showed that 13% of male and 6% of female college students had engaged in sexual intercourse [23]. In contrast, a study conducted in Beijing in 1999 reported that 17% of male and 12% of female university students had experienced premarital sex [24]. Moreover, in a study of young women in Shanghai who received medical examination prior to marriage, 76% reported having had sexual intercourse and 27% had aborted a pregnancy [25].

Furthermore, few studies have explored factors that influence young people's premarital sexual initiation in the Chinese context. Therefore, using baseline data from a community-based comprehensive sex education program in Shanghai, the present study was designed to address four primary research questions: (a) What are out-of-school youth's attitudes towards premarital sex and pregnancy, and their level of sex-related knowledge? (b) Do male and female out-of-school youth communicate with their parents and peers on sex-related matters? (c) How prevalent are premarital sexual behaviors and contraceptive use among out-of-school youth? (d) What are the factors that are associated with premarital sexual intercourse among out-of-school youth? This information is important for policy makers and health educators to develop effective and feasible intervention strategies targeting out-of-school youth who are at increased risk for HIV/STI infection and unplanned pregnancy.

## Methods

### Study sites and participants

The study was carried out from May 2000 to January 2002 in two comparable towns in a suburban district of Shanghai. The original study was a two-arm sex education intervention program with a quasi-experimental design. We selected two typical suburban towns that have a good family planning program network and qualified health providers to administer the program as our research site. The sex education program provided unmarried youth with knowledge about sexual health, contraception, and HIV/AIDS prevention; it also included the distribution of free contraceptives in the intervention town. The selection criteria have been described elsewhere [26].

In May 2000, unmarried youth aged 15–24 who were not planning to marry in the coming year and were willing to participate were enrolled in the study. Family planning workers contacted local authorities (e.g., schools, factories, and community leaders) to develop a list of names of all eligible youth in their villages and to invite them to participate in this study. Initially, we invited 2,362 eligible youth (e.g., 970 in-school and 1,392 out-of-school youth), 2,227 youth (e.g., 923 in-school and 1304 out-of-school youth) agreed to participate in this study and completed a baseline survey. This analysis is based on data from 1304 out-of-school youth (801 males and 503 females) at baseline survey.

### Survey procedure

Participants initially identified by local family planning workers were invited to participate in the survey. A structured questionnaire was administered to all participants who provided written informed consent (parental consent was also obtained for youth less than 18 years). The questionnaire was pre-tested among 30 unmarried youth to ascertain that the content and language were appropriate for the study population. The survey was anonymous and conducted in a private environment (e.g., community meeting rooms). Trained researchers provided explanations and instructions for completing the surveys, and were available to assist participants with any problems they experienced in understanding the questionnaire. Investigators reviewed all questionnaires for completeness and consistency. Responses to open-ended questions were grouped according to the frequency with which they occurred and then coded for analysis according to assigned categories. The study protocol was approved by the Scientific and Ethical Review Group, Department of Reproductive Health and Research, World Health Organization.

The questionnaire collected information on participants' sex, age, education, current occupation, family economic status (categorized as poor, average or rich), family structure, parents' discipline (strict, general or relaxed), feelings about family members (very good, good, less than good), dating status, parent-youth communication on sex related matters, attitudes toward premarital sex and pregnancy, premarital sexual activities, contraceptive use, sexual coercion and pregnancy involvement. The survey also contained questions regarding sexual physiology, contraceptive methods, and HIV/STI transmission, symptoms, and prevention knowledge. Due to low frequencies in some response options, the responses to parental discipline, education, and occupation were combined into two, three, and four categories in statistical analysis, respectively (See Table 1).

**Table 1:** Socio-demographic characteristics of unmarried out-of-school youth

Characteristics	Total	Male	Female	
N	1304	801	503	
Mean age (M ± SD)	19.5 ± 1.5	19.7 ± 1.5	19.3 ± 1.3	***
Age				
16–18	27.8	25.2	31.8	**
19–21	63.3	64.4	61.4	
22–24	9.0	10.4	6.8	
Education				
Junior high or lower	45.4	53.2	33.0	***
Senior high	48.7	42.2	59.0	
College or higher	5.9	4.6	8.0	
Occupation				
Factory worker	63.6	63.8	63.3	***
Seeking employment	24.9	26.9	21.7	
Restaurant worker	4.7	5.2	3.8	
Teacher/technician/clerk	6.8	4.1	11.2	
Family structure				
Both parents	95.2	95.0	95.6	
Single-parent	4.8	5.0	4.4	
Parent's discipline				
Strict	35.2	33.1	38.7	*
General/relaxed	64.8	66.9	61.3	
Feeling toward family				
Very good	33.7	31.2	37.6	**
Good	41.8	41.7	42.0	
Less than good	24.5	27.1	20.5	
Family economic status				
Rich	6.4	5.7	7.5	*
Average	83.4	82.3	85.4	
Poor	10.2	12.0	7.1	
Dating	31.9	29.3	36.0	*

Note: *P < 0.05, **P < 0.01, ***P < 0.001.

### Measures

The primary variables of interest were sex-related knowledge, attitudes towards premarital sex and pregnancy, rates of sexual intercourse, frequency of contraceptive use, and youth's experiences of sexual coercion and/or pregnancy. Premarital sex was indicated by a dichotomous variable – ever had sex (1) or not (0). Hugging, kissing, and fondling were coded as a dichotomous variable (yes/no). Similarly, sexual coercion reflects whether or not the youth ever forced a partner into sex or was forced into sex by a partner. Contraceptive use included how consistently sexually active youth practiced contraception and what method or methods they used. Consistency of use was indicated by a multinomial variable – use every time, use frequently, seldom use and never use. Methods of contraception included: condom, oral contraceptive pills, withdrawal, period, and emergency contraceptive pills. The pregnancy involvement denoted whether or not a youth had conceived or had impregnated a partner.

The survey included 38 items addressing sex-related knowledge regarding sexual physiology, contraceptive methods, and HIV/STI transmission and prevention. Three composite scores were created by adding the number of correct answers to 14 questions on sexual physiology, 12 questions on contraceptive methods, and 12 questions on HIV/STI transmission and prevention. The total number of correct answers to 38 knowledge questions was retained as a sex-related knowledge score with higher scores reflecting increased sex-related knowledge. Subscales for knowledge about physiology, contraception, and HIV/STI are also analyzed. The internal consistency estimates (Cronbach alpha) for the three knowledge subscales was 0.76, 0.84, and 0.78, respectively.

Attitudes about premarital sex were measured using the question "which of the following statements best describes how you feel about having sexual intercourse before marriage?" Five options included: 1) It is alright for people my age to have sex before marriage if both people want to; 2) It is okay for people my age to have sexual intercourse as long as they have fallen in love; 3) Having sexual intercourse before marriage is not a good choice, but I can understand it; 4) Young people who have premarital sex should be condemned for their low social morals; 5) Young people who have premarital sex should be punished. For the purposes of statistical analysis, the responses were grouped into three categories: approve, neutral and disapprove. Response options 1 and 2 were coded to indicate favorable attitudes toward premarital sex, response option 3 indicates neutral attitudes, and response options 4 and 5 indicate unfavorable attitudes toward premarital sex.

Attitudes about premarital pregnancy were measured using the question "which of the following statements best describes how you feel about having a premarital pregnancy?" Four options included: 1) It is a bad thing; 2) It is understandable; 3) It does not matter as long as both people marry; 4) It can promote marriage. For the purposes of statistical analysis, the responses were grouped into three categories: approve, neutral and disapprove. Response option 4 indicated favorable attitudes toward premarital pregnancy, response options 2 and 3 were coded to indicate neutral attitudes, and response option 1 indicated unfavorable attitudes toward premarital pregnancy.

Communication with one's mother or father on sex-related matters was measured using two questions "Have you ever asked your mother (father) sex-related questions?" and "Did your mother (father) initiate discussion about sex?" In multivariate analyses, we coded a positive response to either or both questions referencing mother to indicate that the respondent had communicated with their mother about sexual matters. The same criteria were used to indicate that the respondent had communicated with their father.

### Analysis

We first calculated descriptive statistics of demographic characteristics, attitudes toward premarital sex and pregnancy, parent-youth communication on sex-related issues, premarital sex activities, contraceptive use, sexual coercion, and pregnancy involvement. The gender differences of these variables were tested using Pearson's χ^2^, Cochran-Mantel-Haenszel χ^2 ^test (ordinal variables), and Fisher's exact test (when expected values for one or more cells were less than 5) for categorical variables, and ANOVA for continuous variables.

Second, multivariate logistic regression analyses were performed to identify factors associated with premarital sex for male and female youth. The dependent variable was whether the youth had ever engaged in sexual intercourse (no/yes). These models included socio-demographic factors (e.g., age, education, family structure, feeling to their family, family economic status, and parent's discipline), attitudes towards premarital sex, communication with father or mother on sex-related issues, and dating status. Odds ratio (OR) and its 95% confidence interval (CI) were calculated.

All statistical analyses were performed using the SAS 9.1 statistical software package (SAS Institute Inc., Cary, NC, USA). A significance level of 0.05 was adopted in bivariate comparisons and multivariate analyses.

## Results

### Sample characteristics

As shown in Table 1, 801 (61.4%) of the youth were male. Mean age was 19.5 years, with a range of 16 to 24. Nearly half of the youth had received no more than a junior high school education. Approximately two-thirds of youth were factory workers, and one-fourth were seeking employment. Nearly 5% of the youth lived in a single-parent family and 35% claimed that their parents imposed strict disciplinary control on them. About one-third had very good feelings towards their family and 6% reported that their family was rich. Nearly one-third reported that they were dating someone at the time of the survey.

Compared with male youth, female youth were younger and had a relatively higher education level. A higher proportion of female youth worked as teachers/technicians/clerks (11.2% vs. 4.1%, P < 0.001). Higher proportions of female compared to male youth had very good feelings towards their family (37.6% vs. 31.2%, P < 0.01) and reported that their family was rich (7.5% vs. 5.7%, P < 0.05). Higher proportions of female compared to male youth reported that their parents imposed strict disciplinary control on them (38.7% vs. 33.1%, P < 0.05) and that they were dating someone at the time of the survey (36% vs. 29.3%, P < 0.05).

### Sex-related knowledge and attitudes towards premarital sex

In general, the youth demonstrated a low level of sex-related knowledge (Table 2). On average, youth correctly answered 15 of 38 knowledge items. Youth had a relatively higher level of knowledge about HIV/STI transmission and lower level of knowledge about contraception. On average, youth answered 6 of the 12 knowledge items on HIV/STI transmission and 3 of the 12 knowledge items about contraceptive methods. Male youth were more aware of methods of preventing pregnancy (e.g., withdrawal, condom, spermicide, diaphragm, emergency contraception).

**Table 2:** Sex-related knowledge and attitudes towards premarital sex among unmarried out-of-school youth

Knowledge & Attitudes	Total	Males	Females	
N	1304	801	503	
*Sex-related knowledge*				
Mean score of sex-related knowledge (0–38)	14.9 ± 7.3	15.1 ± 7.6	14.6 ± 6.8	
Sexual physiology (0–14)	5.6 ± 3.2	5.5 ± 3.3	5.8 ± 3.0	*
Contraception (0–12)	3.5 ± 3.0	3.8 ± 3.1	3.1 ± 2.8	***
HIV/STI transmission (0–12)	5.7 ± 2.8	5.8 ± 2.9	5.6 ± 2.6	
*Attitudes towards premarital sex and pregnancy*				
Attitudes towards men's premarital sex^†^				
Favorable	60.8	63.1	56.8	**
Neutral	32.3	31.7	33.3	
Unfavorable	6.9	5.2	9.9	
Attitudes towards women's premarital sex^†^				
Favorable	61.0	63.4	56.8	**
Neutral	31.1	30.5	32.2	
Unfavorable	7.9	6.1	11.0	
Attitudes towards women's premarital pregnancy^†^				
Favorable	8.0	8.6	7.0	***
Neutral	38.8	42.6	32.6	
Unfavorable	53.3	48.8	60.4	

^† ^The statistical significance was tested using Cochran-Mantel-Haenszel Statistics.

*P < 0.05, **P < 0.01, ***P < 0.001.

The majority (60%) of the youth held favorable attitudes towards men's or women's premarital sex and agreed that young people of their age could have premarital sex if they wanted to or if they were in love. Only a small fraction (7%–8%) of youth disapproved of premarital sex and thought young people of their age who had premarital sex should be punished. Nearly one-third of youth held neutral attitudes. They did not approve of premarital sex but said that it is understandable. Although most of the youth held favorable attitudes towards premarital sex, they were less tolerant of premarital pregnancy, with more than half holding unfavorable attitudes. Only 8% endorsed premarital pregnancy because it could promote marriage. Compared with females, males held more liberal attitudes towards premarital sex and premarital pregnancy (Table 2).

### Parent-youth communication on sex-related matters

Table 3 highlights findings regarding parent-youth communication about sex. Few youth of either gender talked to their fathers about sex. A relatively large percentage of female youth talked to their mothers about sex (33–38%). Only 2% to 8% of males talked to their mothers about sex. Both males and females chose their friends as the person with whom they were most likely to talk about sexual matters (Table 3).

**Table 3:** Patterns of communication with parents and peers on sex-related matters among unmarried out-of-school youth

Communication	Total	Males	Females	
N	1304	801	503	
Initiated sex discussion with father	3.5	3.1	4.3	
Father initiated sex discussion with you	6.6	6.9	6.3	
Initiated sex discussion with mother	15.9	2.2	37.6	***
Mother initiated sex discussion with you	17.3	7.7	32.6	***
The person who you mostly likely to talk with about matters of love and sex:				
Friends of same sex	63.0	64.2	61.0	***
Friends of opposite sex	7.7	10.1	3.8	
Father	2.0	2.9	0.6	
Mother	14.4	7.9	24.8	
Brother or sister	4.2	4.4	4.0	
Others (e.g., doctors, teachers, etc.)	8.7	10.5	5.8	

Note: ***P < 0.001.

### Premarital sex and contraceptive use

About 43% of the youth reported having engaged in hugging, one-third in kissing and fondling, and 18% in sexual intercourse. The mean age at first sex was about 20 years. There were no gender differences in engagement in premarital sexual activities except that a higher proportion of male youth reported having engaged in fondling (35% vs. 27%, P < 0.01). The majority of sexually experienced youth (68%) reported that their first sexual partner was their current boy- or girl-friend. A higher proportion of male youth reported that their first sexual partners were their former girlfriends (36% vs. 12%, P < 0.001). About 8% of males reported having forced a partner into sex and 5% of females reported having been forced into sex. Two-thirds of the youth had engaged in sexual negotiation before intercourse. One-quarter of the youth had ever been involved in a pregnancy (Table 4).

**Table 4:** Self-reported sexual activities among unmarried out-of-school youth

Sexual activities	Total	Males	Females	
*All*	1304	801	503	
Sexual activities				
Hugging	43.2	44.2	41.6	
Kissing	34.3	34.6	33.8	
Fondling	31.8	34.7	27.2	**
Sexual intercourse	18.4	18.5	18.3	
Mean age at first sexual intercourse (year)	19.7 ± 1.5	19.7 ± 1.6	19.8 ± 1.2	
*Sexually experienced*^‡ ^(*n*)	(239)	(147)	(92)	
First sexual partner				
Former boy- or girl-friend	26.8	36.1	12.0	***
Current boy- or girl-friend	67.8	58.5	82.6	
General friend or acquaintance	5.4	5.4	5.4	
Forced partner into sex ^†^	4.6	7.5	0.0	**
Was forced into sex ^†^	4.6	4.1	5.4	
Engaged in sexual negotiation before intercourse	66.5	70.8	59.8	
Was involved in a pregnancy	25.8	27.9	22.5	

Note: ^† ^The statistical significance was tested using Fisher's Exact Test; ^‡^: one youth had a missing value on sexual experience. **P < 0.01, ***P < 0.001.

As shown in Table 5, among youth who reported coital experience, frequency of contraceptive use did not differ between males and females. Nearly 40% used a contraceptive method all or most of the time, while 32% used occasionally and 29% never used a contraceptive method. Nearly two-thirds of the youth reported that they and their partner both decided to use a contraceptive method.

**Table 5:** Contraceptive use among sexually experienced out-of-school youth

Contraceptive use	Total	Males	Females	
Frequency of contraceptive use (n)	(239)	(147)	(92)	
Used every time	21.3	19.7	23.9	
Used frequently	18.0	19.1	16.3	
Used occasionally	31.8	33.3	29.4	
Never used	28.9	27.9	30.4	
Whose decision for using contraceptives (n)	(170)	(106)	(64)	
Myself	19.4	23.6	12.5	
Partner	15.3	17.0	12.5	
Both	65.3	59.4	75.0	
Contraceptive methods being used (n)	(170)	(106)	(64)	
Condom	72.9	75.5	68.8	
Oral contraceptive pills	37.6	42.4	29.7	
Withdrawal	35.9	35.9	35.9	
Period	24.1	29.3	15.6	*
Emergency contraceptive pills	7.1	10.4	1.6	*
Reasons for not consistently using contraceptives (n)	(188)	(118)	(70)	
Occasional sex could not lead to a pregnancy	32.8	30.9	35.9	**
Was too shy to buy or obtain contraceptives	19.1	22.2	14.1	
Had no knowledge about contraception	17.2	21.4	10.3	
Sexual intercourse happened unexpectedly	12.8	10.3	16.7	
Contraceptives influence sexual pleasure or mood	9.8	11.1	7.7	
Others	8.3	4.0	15.4	

Note: *P < 0.05, **P < 0.01.

Condom use was the most popular method of contraception used by the youth (73%), followed by oral contraceptive pills (38%) or withdrawal (36%). Nearly one quarter of the youth reported having used menstrual timing ("safe period") and 7% used emergency contraceptive pills. One-third of the youth reported that they did not consistently use a contraceptive method because they thought occasional sex could not lead to a pregnancy, 19% emphasized that they were too shy to buy or obtain contraceptive methods, and 17% said they knew nothing about contraception. Nearly 10% youth believed contraceptives might influence their sexual pleasure and mood.

### Factors associated with premarital sexual intercourse

The results of the logistic regression analysis revealed that age, education level, family structure, parent's discipline, and attitudes towards premarital sex, parent-youth communication on sex-related issues and dating were significantly associated with youth premarital sex (Table 6). For male youth, older age, more relaxed parental discipline, higher level of sex-related knowledge, favorable attitudes, communicating with father regarding sex-related matters, and dating were associated with premarital sex. For female youth, higher education, single-parent family, favorable attitudes, communicating with mother regarding sex-related issues and dating were significantly associated with premarital sex. Youth's feeling towards their family and their family economic status was not associated with either male or female youth's premarital sexual initiation.

**Table 6:** Association between various characteristics and premarital sexual intercourse among out-of-school youth

Variables	Male (n = 801)		Female (n = 503)	
		
	OR	95% CI	OR	95% CI
Age (year)				
Continuous variable	1.20	1.02~1.42	1.04	0.81~1.34
Education				
Junior high or lower (ref)				
Senior high or above	0.75	0.46~1.20	0.22	0.11~0.45
Family structure				
Both parents family (ref)				
Single parent family	0.71	0.23~2.22	4.84	1.21~19.37
Feeling to their family				
Very good (ref)				
Good	0.91	0.53~1.58	0.74	0.35~1.55
Less than good	1.33	0.73~2.42	1.70	0.71~4.08
Family economic status				
Poor (ref)				
Average	0.96	0.48~1.94	0.62	0.16~2.49
Rich	1.93	0.65~5.74	0.64	0.10~4.12
Parent's discipline				
Strict (ref)				
General or relaxed	1.91	1.15~3.18	0.68	0.33~1.39
Sex-related knowledge	1.11	1.02~1.22	1.01	0.88~1.16
Attitude towards premarital sex				
Unfavorable (ref)				
Neutral	1.60	0.51~6.27	2.71	0.77~9.57
Favorable	3.52	1.05~13.10	3.38	1.03~11.17
Having communicated with father about sex-related issues				
No (ref)				
Yes	2.29	1.03~5.28	0.55	0.17~1.80
Having communicated with mother about sex-related issues				
No (ref)				
Yes	1.54	0.68~3.49	2.49	1.17~5.32
Dating				
No (ref)				
Yes	13.80	8.62~22.11	38.68	15.89~94.15

Notes: ref = reference; CI = confidence interval; OR = Odd Ratio.

## Discussion

The majority of out-of-school youth in this study indicated favorable attitudes about premarital sex, although acknowledged the potential risk of engaging in premarital sexual intercourse. Although a higher proportion of male compared to female youth held favorable attitudes towards premarital sex, there was no gender difference in rates of premarital sexual intercourse. This finding is inconsistent with previous studies in China reporting that male college students were significantly more likely to have experienced sexual intercourse than females [6,24]. Only one-fifth of sexually active youth used a contraceptive method all the time. The rate of consistent condom use was even lower among this group. Premarital pregnancy was reported by one quarter of sexually active youth. In addition, sexual coercion was not rare among male out-of-school youth. These findings demonstrate that out-of-school youth are at great risk for HIV/STI and premarital pregnancy, suggesting the importance of targeting prevention efforts towards them.

Rates of sexual experience reported by the out-of-school youth were higher than those reported by university students in China [22,27], although they are similar in age. These findings suggest that the school environment may offer a context which discourages sexual activity [28]. In China, many high schools or universities have regulations that limit intimate relationships between students of opposite sex in school. Out-of-school youth have had more opportunities to engage in sexual activity, as their social life exists in a context that is more relaxed than it was when they were in high school or university. Further, by custom in some suburbs of Shanghai, unmarried young men and women can live together after being engaged [29].

This group of out-of-school youth generally had a low level of knowledge about sexual matters, particularly with regard to the issue of contraception. Sex education for adolescents in public schools in China provides little information on contraception [30]. The low level of knowledge of HIV/STI transmission and contraception and the fact that a large proportion of youth used the rhythm and/or withdrawal suggest the areas that need to be addressed in the development of sex education and HIV prevention programs. Consistent with a previous study [31], male youth seemed more aware of methods of preventing pregnancy, especially male contraception methods (i.e., withdrawal and condom).

Data in the current study showed that male youth whose parents did not apply strict discipline and female youth living with a single parent were more likely to have experienced sexual intercourse, suggesting the importance of family context in sexual initiation. Dating and favorable attitudes towards premarital sex were prominent factors associated with premarital sex. Our study indicated that male youth who communicated with fathers, and female youth who communicated with mothers on sex-related matters were more likely to engage in premarital sex, which is inconsistent with some previous studies demonstrated a protective relationship between parent-youth communication and premarital sexual behavior [32,33].

This study has several potential limitations. First, we did not use a probabilistic sampling method to select the study population as this study is part of the baseline survey of a community-based comprehensive sex education program. Generalization of findings from this study to out-of-school youth in other areas is limited. Second, the bias introduced by under-reporting is possible as premarital sex is a sensitive issue and may be considered socially unacceptable in mainly Chinese cultural settings [34]. However, by ensuring privacy during the completion of the questionnaire and using the anonymous self-administered survey, an attempt was made to minimize this bias.

## Conclusion

To the best of our knowledge, this is the first study which examines sexual attitudes and behavior among out-of-school youth in China. The results of this study have implications for future HIV prevention programs. First, out-of-school youth should be targeted with HIV prevention efforts as they are at increased risk for HIV/STI. Second, they should be educated about contraception and HIV/STI transmission, communication and sexual negotiation skills, and condom use skills. Third, parental influences should be considered when designing interventions to postpone adolescents' sexual activities. To discourage their sexual initiation, adolescents should be targeted with prevention interventions when they are in middle schools.

## Competing interests

The author(s) declare that they have no competing interests.

## Authors' contributions

BW participated in the design of the study and acquisition of data, performed the statistical analysis, and drafted the manuscript. XL, BS, and VK helped draft the manuscript, and made contributions to the revisions. SN, IS, and RT participated in data analysis and critically read the manuscript.

## Pre-publication history

The pre-publication history for this paper can be accessed here:


